# Extract of Wax Gourd Peel Prevents High-Fat Diet-Induced Hyperlipidemia in C57BL/6 Mice via the Inhibition of the PPAR**γ** Pathway

**DOI:** 10.1155/2013/342561

**Published:** 2013-02-25

**Authors:** Ming Gu, Shengjie Fan, Gaigai Liu, Lu Guo, Xiaobo Ding, Yan Lu, Yu Zhang, Guang Ji, Cheng Huang

**Affiliations:** ^1^School of Pharmacy, Shanghai University of Traditional Chinese Medicine, Shanghai 201203, China; ^2^College of Horticulture and Landscape Architecture, Southwest University, Chongqing 400716, China; ^3^Key Laboratory of Horticulture Science for Southern Mountainous Regions, Ministry of Education, Chongqing 400715, China; ^4^Institute of Digestive Disease, Longhua Hospital, Shanghai University of Traditional Chinese Medicine, Shanghai 201203, China

## Abstract

Wax gourd is a popular vegetable in East Asia. In traditional Chinese medicine, wax gourd peel is used to prevent and treat metabolic diseases such as hyperlipidemia, hyperglycemia, obesity, and cardiovascular disease. However, there is no experimental evidence to support these applications. Here, we examined the effect of the extract of wax gourd peel (EWGP) on metabolic disorders in diet-induced C57BL/6 obese mice. In the preventive experiment, EWGP blocked body weight gain and lowered serum total cholesterol (TC), low-density lipoprotein cholesterol (LDL-c), liver TG and TC contents, and fasting blood glucose in mice fed with a high-fat diet. In the therapeutic study, we induced obesity in the mice and treated with EWGP for two weeks. We found that EWGP treatment reduced serum and liver triglyceride (TG) contents and fasting blood glucose and improved glucose tolerance in the mice. Reporter assay and gene expression analysis showed that EWGP could inhibit peroxisome proliferator-activated receptor **γ** (PPAR**γ**) transactivities and could decrease mRNA levels of PPAR**γ** and its target genes. We also found that HMG-CoA reductase (HMGCR) was downregulated in the mouse liver by EWGP. Our data suggest that EWGP lowers hyperlipidemia of C57BL/6 mice induced by high-fat diet via the inhibition of PPAR**γ** and HMGCR signaling.

## 1. Introduction

Hyperlipidemia is a serious epidemic disease involving lipid metabolism disorder and is a key pathogenic factor resulting in diabetes and cardiovascular diseases [1, 2]. The increased prevalence of hyperlipidemia has been an epidemic public and economic problem. Pharmacotherapy is the primary way of treating dyslipidemia at present, with prescription drugs, such as statins, fibrates, nicotinic acids, and bile acid sequestrants, dominating the main drug market [3]. Although clinical trials have repeatedly proved that these drugs are effective in modulating hyperlipidemia, side effects, such as toxicity of the liver and kidneys, cannot be ignored.

Dietary therapy for dyslipidemia is an attractive way for patients to manage this condition. In China, many medicinal herbs, such as Coptis rhizome, ginseng, *Astragalus mongholicus,* and green tea, are used in formulas for the prevention and treatment of dyslipidemia [4–6]. Food-medicine dual plants are an important part of traditional Chinese medicine. Several food-medicine duals, such as bitter melon, ginger, celery, *Citrus maxima*, hawthorn, and red kojic rice, have been proven to be of benefit in dyslipidemia [7].

Wax gourd, also known as winter melon, white gourd, or fuzzy gourd, is the only member of the genus* Benincasa*, originally cultivated in Southeast Asia, and is now an important vegetable in East Asian countries. In traditional Chinese medicine, wax gourd is used as a dietary therapy for anti-inflammatory, antioxidant, and antiangiogenesis purposes and also for treating nociception, pyretic diarrhea, and ulcers [8–10]. In addition, wax gourd is used to treat obesity, hyperglycemia, hyperlipidemia, and as a protection against kidney diseases in China and other East Asian countries [11, 12]. Similarly, the seed extract of wax gourd and wax gourd peel also have ameliorate metabolic disorders, antiangiogenesis, and anticancer effects [13]. However, little experimental data could be found to support the effects of wax gourd peel (WGP) on alleviating these metabolic disorders.

Peroxisome proliferator-activated receptors (PPARs) are nuclear receptor transcription factors and have been identified as drug targets for metabolic disorders. These receptors are lipid and glucose metabolism sensors and are present in adipocytes, liver, and muscle [14]. Previous studies have shown that the inhibition of PPAR*γ* could reduce fat and body weight and improve insulin resistance via the modulation of genes related to lipid and glucose metabolism [15–17]. Many herbal or natural medicines, that act as modulators of PPARs, have been reported to block intracellular lipid accumulation and lipogenesis and to improve insulin resistance [18, 19]. For example, Gong et al. reported that tanshinone IIA in *Salvia miltiorhiza* can be used to treat obesity through PPAR*γ* antagonism [20]. In addition, Goldwasser et al. reported that naringenin from grapefruit could regulate hepatic lipid metabolism by influencing the activity of PPARs [21]. Huang et al. meanwhile reported that berberine from *Coptis rhizome* could inhibit intracellular lipid accumulation in 3T3-L1 cells by the PPAR*γ* pathway [22]. Here, we show that extract of wax gourd peel (EWGP) may prevent the development of hyperlipidemia and insulin resistance in high-fat diet-fed C57BL/6 mice via inhibition of the transactivities of PPAR*γ* and reduction in the expression of its downstream genes.

## 2. Materials and Methods

### 2.1. Chemicals and Diet

WGP (Shanghai Lei Yun Shang Medicinal Materials Co.) was extracted with 75% ethanol. The extract of WGP (EWGP) was concentrated at 40°C with a rotary evaporator under reduced pressure, freezedried to a powder, and dissolved in dimethyl sulfoxide (DMSO). Rosiglitazone (Ros) and WY14643 were purchased from Sigma-Aldrich (St. Louis, MO, USA). High-fat diets (60% of calories derived from fat) and chow diet (10% of calories derived from fat) were purchased from Research Diets (D12492, D12450B).

### 2.2. Animals and Treatment

The animal protocols used in this study were approved by Shanghai University of Traditional Chinese Medicine. Female C57BL/6 mice were purchased from the SLAC Laboratory (Shanghai, China). All animals were kept under controlled temperature (22-23°C) and on a 12-h light, 12-h dark cycle. For the preventive treatment, the six-week-old female C57BL/6 mice were randomly divided into three groups according to body weight: chow (10% of calories derived from fat, *n* = 7), high-fat (HF, 60% of calories derived from fat, *n* = 7), and high-fat plus 1% EWGP (EWGP was powdered and mixed into HF diet, *n* = 7). Mice were treated for 8 weeks. Twenty-four-hour food intake amount was measured by recording the difference in weight between the food put into the cage and that remaining at the end of twenty-four hours.

For the therapeutic treatment, six-week-old mice were fed with a high-fat diet for 12 weeks to induce obesity. The obese animals were then randomly separated into either the HF or EWGP group, with the latter group being treated as in the preventive treatment. The chow-control mice continued to be fed the chow diet throughout the experiment. The mice were treated in this way for 2 weeks. Body weight and food consumption were recorded every 2 days.

### 2.3. Intraperitoneal Glucose Tolerance Test

At the end of the treatment, mice were fasted overnight (12 h). The baseline glucose values (0 min), prior to injection of glucose (1 g/kg body weight), were measured by means of collecting blood samples from the tail vein. Additional blood samples were collected at regular intervals (15, 30, 60, and 90 min) for glucose tolerance tests.

### 2.4. Serum Chemistry Analysis

The mice were fasted overnight and anesthetized, and cardiac blood was taken. Serum triglyceride (TG), total cholesterol (TC), HDL cholesterol (HDL-c), and LDL cholesterol (LDL-c) were measured using a Hitachi 7020 Automatic Analyzer (Hitachi Ltd., Tokyo, Japan) using 100 *μ*L of heart blood serum.

### 2.5. Determination of Intracellular Triglyceride and Cholesterol Levels

HepG2 cells were cultured in Dulbecco's Modified Eagle Media (DMEM) containing normal glucose (5.5 mmol/L D-glucose) supplemented with 10% fetal bovine serum (FBS, Hyclone, Logan, UT), 100 units/mL penicillin, and 100 *μ*g/mL streptomycin. Cells were incubated in a humidified atmosphere of 5% CO_2_ at 37°C. Cells were passaged and seeded on a 12-well plate. The cell growth was quenched in serum-free DMEM, containing normal glucose, overnight when cells had grown to 70% confluence. The cells were then incubated in high glucose (30 mmol/L D-glucose) and treated with EWGP for 24 hours after serum depletion. Cells without EWGP treatment were used as a high glucose-induced lipid accumulation control. HepG2 cells were homogenized in lysis buffer (20 mM Tris-HCl, pH 7.5, 150 mM NaCl, 1% Triton), and protein concentrations in cell lysates were measured using a Bio-Rad Protein Assay Kit. Cell lysates were then mixed with an equal volume of chloroform, followed by centrifugation at 14,000 rpm for 15 min at 4°C. The chloroform layer was separated, dried, and resuspended in 50 *μ*L of isopropyl alcohol to measure the intracellular triglyceride and total cholesterol contents as previously described [23]. Intracellular lipid contents in HepG2 cell lysates were expressed as micrograms of lipid per milligram of cellular protein.

### 2.6. Liver Lipid Content Analysis

The liver tissues were weighed and homogenized in lysis buffer (20 mM Tris-HCl pH 7.5, 150 mM NaCl, 1% Triton) and extracted with an equal volume of chloroform. The chloroform layers were dried and dissolved in isopropyl alcohol to measure lipid levels as described [23].

### 2.7. Quantitative Real-Time PCR Analysis in Animal Tissues and HepG2 Cells

The quantitative real-time PCR assay was performed as previously described [22]. Briefly, total RNA was extracted from the liver samples using spin columns (Qiagen, Germany) according to the manufacturer's instructions. Genomic DNA contamination was removed by using DNase I. The first strand cDNA was synthesized with a cDNA Synthesis Kit (Fermentas, Madison, WI). An ABI StepOnePlus real-time PCR system (Applied Biosystems, USA) was used to analyze the gene expression levels. The cDNA was denatured at 95°C for 10 min followed by 40 cycles of PCR (95°C, 15 s; 60°C, 60 s). The primers used in the experiments are listed in Table 1. *β*-actin was used as an internal control to normalize all the mRNA levels.

HepG2 cells were grown in a 12-well plate to 80% confluence with high glucose DMEM containing 10% FBS at 37°C in 10% CO_2_, and then EWGP was added to the medium at 200 *μ*g/mL for 24 hours. Total RNA was extracted, and genes expression analysis was performed as described above. The primers used in the cellular experiments are listed in Table 2. *β*-actin was used as an internal control to normalize all the mRNA levels.

### 2.8. Transfection of Cultured Cells and Reporter Assays

The reporter assay was performed as previously described [20, 22]. The expression plasmid pCMXGal-mPPAR*α*, *γ*-LBD, and the Gal4 reporter vector MH100 × 4-TK-Luc were cotransfected with a reporter construct so that 1 *μ*g of the relevant plasmid combined with 1 *μ*g of reporter plasmids and 0.1 *μ*g of pREP7 (*Renilla* luciferase) reporter to normalize transfection efficiencies. The transfection mixture, which contained 10 *μ*g of total plasmids and 15 *μ*L lipofectamine 2000 (Roche) per mL of DMEM, was added to HEK293T cells for 24 h and then removed. The PPAR*α* and PPAR*γ* agonists (WY14643 and rosiglitazone), or EWGP, were added to fresh media and the cells were incubated for another 24 h to determine luciferase activity. We conducted the luciferase reporter assays using the Dual-Luciferase Reporter Assay System (Promega, USA), and *Renilla* luciferase activity was assayed to normalize transfection efficiencies. All the transfection experiments were performed in triplicate and repeated at least three times independently.

### 2.9. Statistical Analysis

Data analyses were performed using the statistical program SPSS 12.0 for Windows. All data were presented as means ± SE. Statistical analysis was performed using one-way analysis of variance (ANOVA). Differences were considered as significant (∗), *P* < 0.05 or not significant (NS), *P* > 0.05.

## 3. Results

### 3.1. EWGP Prevents Metabolic Disorders Induced in C57Bl/6 Mice by a High-Fat Diet

**Figure 1:**
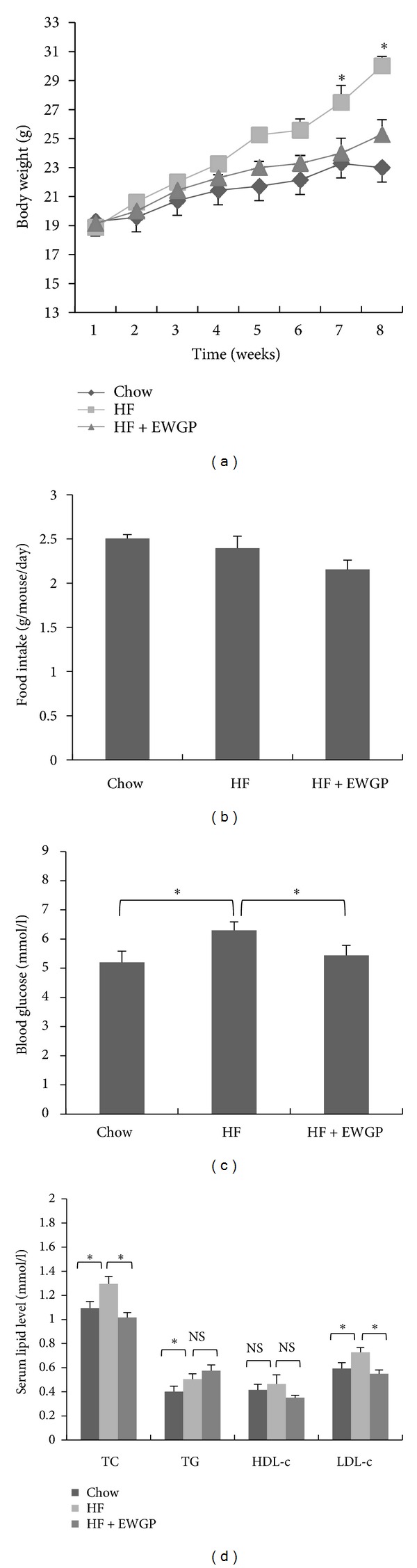
EWGP prevents metabolic disorders induced in C57BL/6 mice by a high-fat diet. (a) Body weight gain. (b) Diet consumption. (c) Fasting glucose levels. The mice were fasted for 12 h, and tail vein blood was used to measure the blood glucose levels. (d) Serum TC, TG, HDL-c, and LDL-c content. The data are shown as means ± SE. *n* = 7 for all groups. *P < 0.05. NS: Not significant.

To test whether EWGP blocks diet-induced body weight gain and dyslipidemia, C57BL/6 mice were fed a high-fat (HF) diet or HF diet mixed with 1% EWGP for 8 weeks. The mice fed HF diet displayed a larger body weight gain compared to the chowdiet after 8 weeks of treatment. The EWGP supplemented HF diet meanwhile blocked the body weight gain of the mice (Figure 1(a), *P* < 0.05). The food intake amount was not significantly different between the HF and EWGP groups (Figure 1(b)), indicating that the lower body weight in EWGP-treated mice did not result from decreased calorie intake. We then assayed blood glucose and lipid levels of the mice.  Figure 1(c) shows that the fasting blood glucose levels in HF-fed mice were markedly higher than that in chow-control mice, while EWGP treatment significantly lowered the fasting glucose levels in the mice (*P* < 0.05). The HF-fed mice showed higher levels of serum TC, TG, and LDL-c when compared to chow-control mice (Figure 1(d), *P* < 0.05). EWGP-treated mice had significantly reduced serum TC and LDL-c levels compared to HF-control mice. However, TG and HDL-c levels were not significantly changed (Figure 1(d)). These results suggest that EWGP could block the body weight gain and metabolic disorders induced by a high-fat diet in C57BL/6 mice.

### 3.2. EWGP Improves Glucose Tolerance in Obese Mice

**Figure 2:**
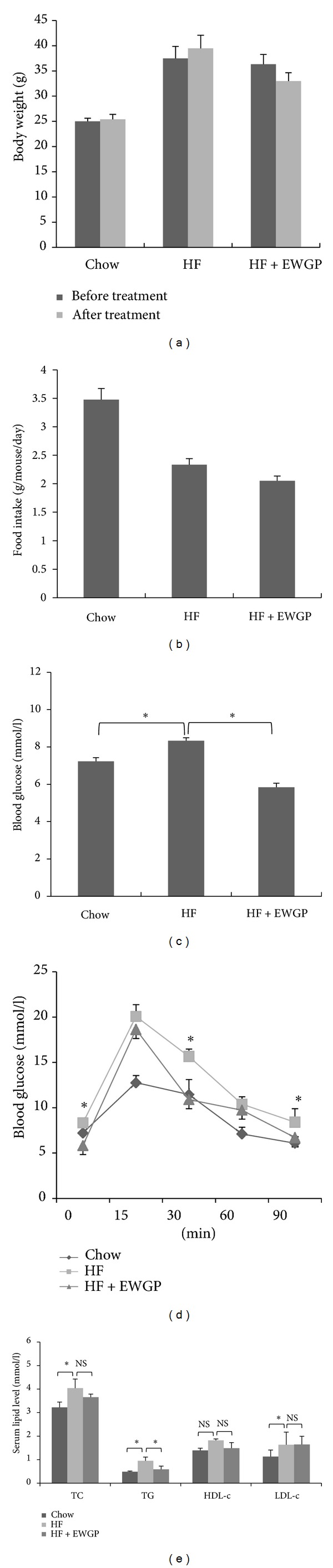
Therapeutic effects of EWGP on obese C57BL/6 mice. (a) Body weight. (b) Diet consumption. (c) Fasting glucose levels. The mice were fasted for 12 h, and tail vein blood was used to measure the blood glucose levels. (d) Intraperitoneal glucose tolerance test (IPGTT). (e) Serum TC, TG, HDL-c, and LDL-c content. The data are shown as means ± SE. *n* = 7 for all groups. *P < 0.05. NS: Not significant.

To study whether EWGP could alleviate metabolic disorders in obese mice, we fed mice with a HF diet (HFD) for 12 weeks to induce obesity. The obese mice were then grouped and fed with either HFD alone or HFD mixed with 1% EWGP for 2 weeks. Results showed that EWGP treatment did not significantly reduce body weight of the mice (Figure 2(a)). The food intake amount was not significantly different between the HFD and EWGP groups (Figure 2(b)). It is well known that insulin resistance commonly coexists with obesity. To understand whether EWGP improved insulin resistance *in vivo*, we measured the fasting blood glucose levels and glucose tolerance of the mice. The HF-fed mice exhibited higher fasting blood glucose levels when compared to chow-control mice, while the EWGP-treated group showed lower fasting glucose levels than HF-fed mice (Figure 2(c), *P* < 0.05). We then assayed intraperitoneal glucose tolerance and found that EWGP significantly improved glucose tolerance in the obese mice at 30 and 90 min following intraperitoneal injection of glucose (Figure 2(d), *P* < 0.05).

Next, we assayed the serum lipid levels of the mice. The HF-fed mice showed higher levels of serum TC, TG, and LDL-c when compared to those in chow-control mice (Figure 2(e), *P* < 0.05). EWGP treatment significantly lowered serum TG levels when compared to HF-feeding (Figure 2(e), *P* < 0.05). However, TC, HDL-c, and LDL-c levels remained unchanged. Taken together, the results indicate that EWGP could improve glucose and lipid metabolism in the obese mice.

### 3.3. Effect of EWGP on Lipid Accumulation in HepG2 Cells

**Figure 3:**
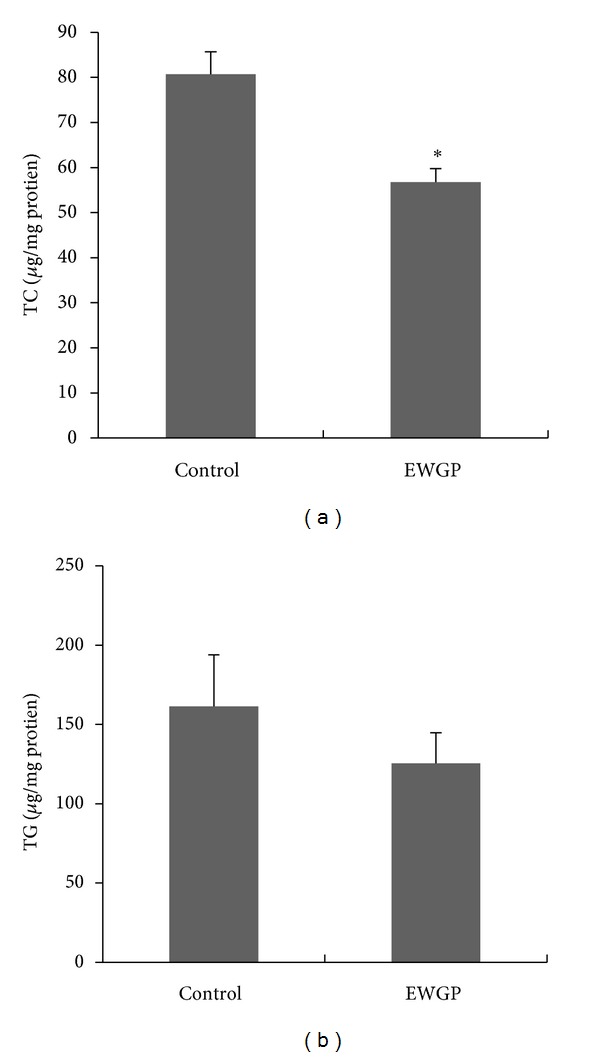
EWGP blocks high-glucose-induced lipid accumulation in HepG2 cells. (a) Intracellular cholesterol levels in HepG2 cells. (b) Intracellular triglyceride levels in HepG2 cells. The data are shown as means ± SE. *n* = 4 for all groups. *P < 0.05 versus control group.

To confirm the lipid-lowering effect of WGP, we treated human liver carcinoma HepG2 cells, a lipid metabolism cell model, with EWGP to analyze whether EWGP could ameliorate intracellular lipid metabolism. The HepG2 cells were cultured with the high-glucose medium to induce lipid accumulation, and then EWGP (200 *μ*g/mL) was added to treat the cells for 24 hours. The results showed that EWGP notably lowered intracellular cholesterol levels (Figure 3(a), *P* < 0.05) when compared with the vehicle control. However, we did not observe any significant effects of EWGP on intracellular triglyceride levels of HepG2 cells (Figure 3(b)), suggesting that EWGP may have an inhibitory effect on the intracellular cholesterol accumulation.

### 3.4. EWGP Improves Lipid Accumulation in the Liver of C57BL/6 Mice

**Figure 4:**
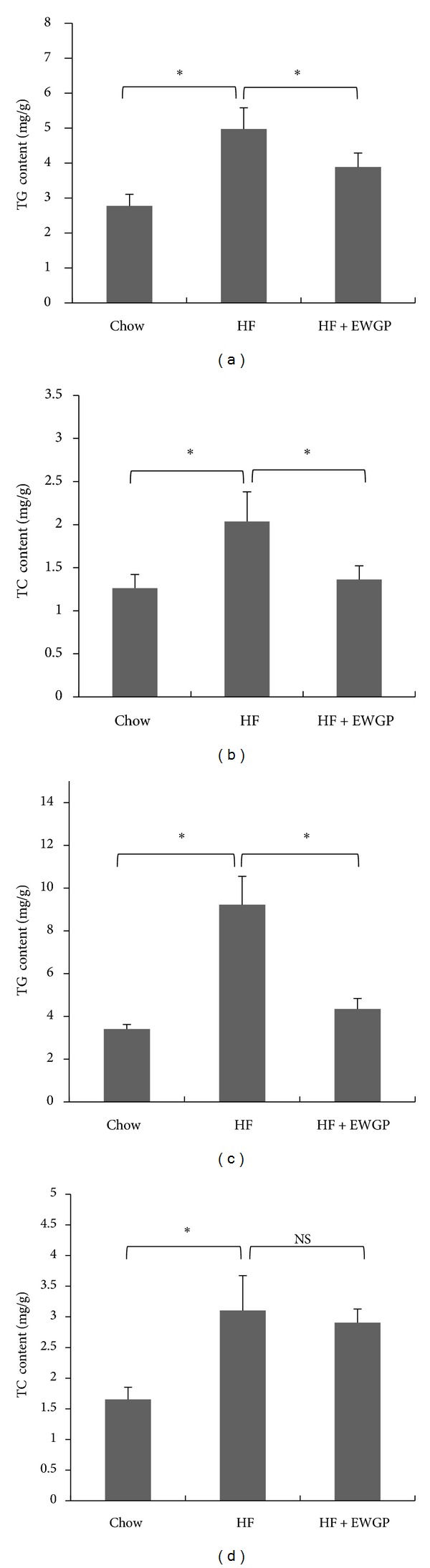
EWGP improves lipid accumulation in the liver of high-fat diet-induced C57BL/6 mice. TG (a) and TC (b) contents in the mouse liver of preventive treatment. TG (c) and TC (d) contents in the mouse liver of therapeutic treatment. The data were shown as means ± SE. *n* = 7 for all groups. *P < 0.05. NS: No significance.

Liver is an important organ for lipid metabolism. We measured TG and TC contents in the mouse liver of the preventive and therapeutic experiment. In the preventive treatment, TG and TC levels in the HF diet-fed mice were markedly increased when compared to those in the chow diet control mice, whereas EWGP treatment significantly decreased TG and TC accumulation in the livers of the HF diet-fed mice (Figures 4(a) and 4(b), *P* < 0.05). In therapeutic treatment, the TG and TC levels in the obese mouse liver were markedly higher than those in the control mouse liver, while EWGP treatment significantly lowered TG contents in the obese mouse liver (Figure 4(c), *P* < 0.05). However, TC content in EWGP-treated mouse liver was not significantly changed compared to that in obese control mice (Figure 4(d)).

### 3.5. EWGP Inhibits PPAR Signaling

**Figure 5:**
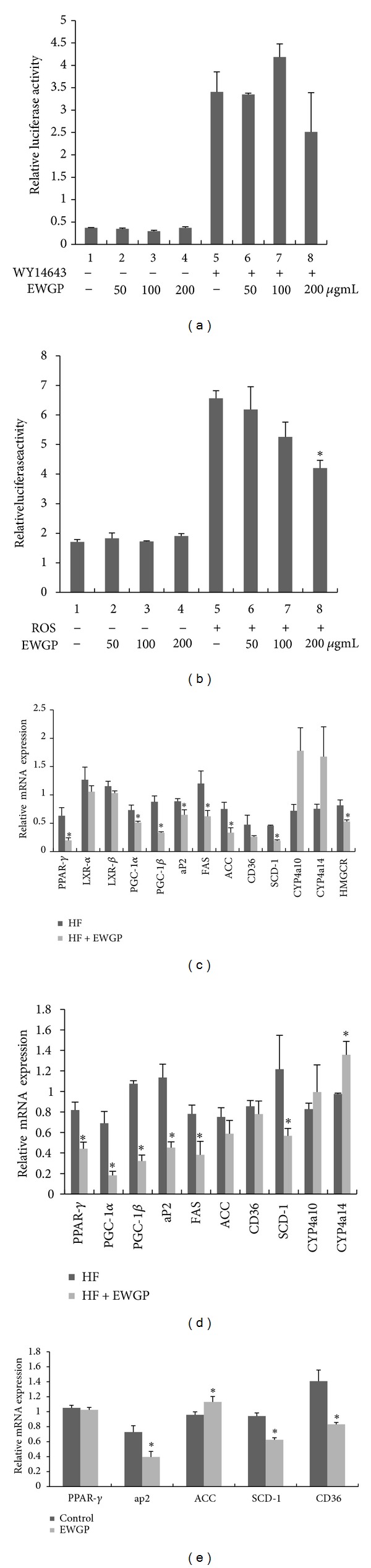
EWGP regulates PPAR*γ* transcription activity and genes related to glucose and lipid metabolism. ((a) and (b)) The transcription activity of PPAR*α*, *γ*. GAL4-DBD-LBD expression plasmids and a GAL4-responsive luciferase reporter were cotransfected into HEK293T cells for 24 h and treated with the PPAR*α* and PPAR*γ* agonists WY14643 and rosiglitazone (20 *μ*M) and EWGP (50, 100, and 200 *μ*g/mL) for another 24 h. The relative luciferase activities were measured by comparison to *Renilla* luciferase activities. The results represent three independent experiments, and data are presented as means ± SE. (c) The relative gene expression levels in the liver tissues from EWGP-treated and HF-fed mice in the preventive treatment. (d) The relative gene expression levels in the liver tissues from EWGP-treated and HF-fed mice in the therapeutic treatment. *β*-actin was used as an internal control for normalizing the mRNA levels. Data are presented as means ± SE for seven mice per group. *P < 0.05 versus HF group. (e) The relative gene expression levels in EWGP-treated and control HepG2 cells. *β*-actin was used as an internal control for normalizing the mRNA levels. Data are presented as means ± SE for six samples per group. *P < 0.05 versus control group.

PPARs are important regulators of lipid and glucose homeostasis through the modulation of the expression of important downstream target genes. We determined PPAR*α*, *γ* transcription activities with a reporter assay system. The results showed that EWGP did not influence PPAR*α* transcription activity (Figure 5(a)), whereas it markedly inhibited PPAR*γ* transcription activity at concentrations of 200 *μ*g/mL (Figure 5(b), *P* < 0.05). To confirm this effect, we evaluated the *in vivo* effect of EWGP on the expression of PPAR*γ* target genes by analyzing their mRNA expression levels in liver tissues from EWGP-treated and HF-fed control mice. The mRNA levels of PPAR*γ* and its target genes aP2, FAS, ACC, and SCD-1 were decreased significantly, and the expression of PPAR*γ* coactivators PGC1-*α*, PGC1-*β* was also notably lowered (Figures 5(c) and 5(d), *P* < 0.05), whereas CD36 mRNA was not changed markedly (Figures 5(c) and 5(d)). Then, we assayed the gene expression levels of PPAR*α* target genes, CYP4a10, and CYP4a14 [24]. The results showed that the mRNA levels of CYP4a10 and CYP4a14 were not significantly changed in the preventive treated mouse liver (Figures 5(c) and 5(d)). However the expression of CYP4a14 was increased in the liver of therapeutic-treated mouse (Figure 5(d), *P* < 0.05).

We then analyzed gene expression level in EWGP-treated HepG2 cells. The results showed that EWGP significantly decreased the expression level of PPAR*γ* target genes such as aP2, CD36, and SCD-1 in HepG2 cells (Figure 5(e), *P* < 0.05). These results indicate that EWGP could inhibit PPAR*γ* signaling both *in vivo* and *in vitro*.

### 3.6. EWGP Inhibits the mRNA Level of Hmg-CoA Reductase

HMG-CoA reductase (HMGCR) is a key enzyme in cholesterol synthesis and therefore a drug target for lowering TC and TG. We found that in the liver tissues of EWGP-treated mice, the expression of HMGCR was also downregulated (Figure 5(c), *P* < 0.05), although the expression of its upstream regulators liver X receptor *α* and *β* (LXR*α*, *β*) were not changed in EWGP-treated mouse livers (Figure 5(c)). These results indicate that wax gourd peel could inhibit the expression of HMGCR to improve metabolism disorder.

## 4. Discussion

Wax gourd peel has been used as a food and in dietary medicine for prevention and treatment of lipid and glucose metabolism disorder and obesity for hundreds of years. It has been reported that four triterpenes, two sterols, a flavonoid C-glycoside, an acylated glucose, and a benzyl glycoside have been identified from Wax gourd and WGP [25, 26]. Triterpenes could lower serum cholesterol, beta-lipoproteins, and triglycerides in hyperlipidemic mice [27–29]. However, little experimental data could be obtained regarding its pharmacological mechanisms. In the present study, we provide evidence that WGP prevents metabolic disorder, and especially, ameliorates dyslipidemia in high-fat diet-fed mice. Our results not only showed that EWGP could effectively prevent lipid and glucose metabolic disorders in mice fed with a high-fat diet but also showed that EWGP had therapeutic effects in improving glucose tolerance in the obese mice.

In the preventive treatment, EWGP blocked the body weight gains in EWGP-treated mice compared with HF-fed mice. This effect is not linked to food intake as there was no notable difference in diet consumption between the groups. We also noticed differences in lipid levels between the preventive and therapeutic treatments of the mice. The TC and LDL-c levels in the preventive experiment were significantly lowered, but they were not in therapeutic experiment. This discrepancy may be a result of the shorter duration of the therapeutic treatment. In the preventive therapy, we treated mice for 8 weeks but only 2 weeks for therapeutic treatment. Extension of the therapeutic time may therefore achieve better results.

We found that EWGP inhibited the transactivity of the nuclear receptor transcription factor PPAR*γ* but not PPAR*α*. PPAR*γ* is a nuclear transcription factor, expressed in a wide range of tissues including adipose tissue, liver, and intestine. PPAR*γ* participates in mediating metabolic disorder via numerous downstream adipogenic and lipogenic genes, which are important for adipocyte maturation, lipid accumulation, and insulin-sensitive glucose transport, including FAS, ACC, aP2, SCD-1, and CD36 [15, 16, 30]. FAS and ACC are involved in triglyceride synthesis, and their expression levels are known to increase in steatotic livers of *ob*/*ob *mice [31]. SCD-1 and CD36 are involved in fatty acid uptake and oxidation, and mice that lack SCD-1 are lean and resistant to obesity [31]. As an adipocyte marker, aP2 modulates inflammatory responses and cholesterol ester accumulation, and deficiency in aP2 could protect against atherosclerosis and insulin resistance in the ApoE^−/−^ mice [32–35]. PGC1-*α* and PGC1-*β* are coactivators of PPAR*γ* signaling and have mutual promotion with PPAR*γ*. In the liver, the PGC-1 increases lipogenesis and lipoprotein transport [36, 37]. Although PPAR*γ* agonists such as rosiglitazone and pioglitasone improve insulin resistance and glucose tolerance in obese mice models, previous studies also have shown that inhibition of PPAR*γ* signaling significantly improve metabolic disorders including obesity, dyslipidemia, and diabetes [20, 22]. Therefore, we postulate that effects of EWGP on metabolic disorders may be partly through the inhibition of PPAR*γ* signaling.

Nuclear receptor transcription factor LXRs are important regulators of lipid and glucose homeostasis [38, 39]. Therefore, we also tested the gene expression levels of LXR*α* and *β*. We did not observe any alteration in the mRNA levels of LXR*α* and *β*. However, our data showed EWGP was effective in lowering TC levels in both mouse livers and HepG2 cells. HMG-CoA reductase (HMGCR) is a key enzyme in cholesterol synthesis and is the target of the lipid-lowering drug statin [40]. Decreasing mRNA expression, or levels of the HMGCR protein, could decrease TC levels. We found that EWGP decreased HMGCR mRNA expression, suggesting that EWGP may reduce TC content through the inhibition of HMGCR. Taken together, our data indicate that EWGP may regulate lipid and glucose disorders via the inhibition of PPAR*γ* signaling and mRNA levels of HMGCR.

## 5. Conclusions

In summary, our results provide evidence that EWGP played a role in ameliorating metabolic disorders in high-fat diet-fed mice. Its pharmacological mechanism may result from the suppression of PPAR*γ* signaling and HMGCR. EWGP may be a good choice as a safe dietary strategy for preventing hyperlipidemia and other metabolic disorders. Further investigation is needed to define the mechanisms by which this component protects against metabolic disorders and whether other mechanisms exist also.

## Figures and Tables

**Table 1:** Sequence of the primers used in real-time PCR of the mouse tissue.

Gene	Forward primer	Reverse primer
*β*-Actin	TGTCCACCTTCCAGCAGATGT	AGCTCAGTAACAGTCCGCCTAGA
LXR*α*	GAGTGTCGACTTCGCAAATGC	CCTCTTCTTGCCGCTTCAGT
LXR*β*	CAGGCTTGCAGGTGGAATTC	ATGGCGATAAGCAAGGCATACT
HMGCR	GGGCCCCACATTCACTCTT	GCCGAAGCAGCACATGATCT
FAS	CTGAGATCCCAGCACTTCTTGA	GCCTCCGAAGCCAAATGAG
CYP4a10	GAGTGTCTCTGCTCTAAGCCCA	AGGCTGGGGTTAGCATCCTCCT
CYP4a14	FGAATTGCTGCCAGATCCCACCAGGATC	GTTCAGTGGCTGGTCAGA
PGC-1*α*	TGTTCCCGATCACCATATTCC	GGTGTCTGTAGTGGCTTGATTC
PGC-1*β*	GGGTGCGCCTCCAAGTG	TCTACAGACAGAAGATGTTATGTGAACAC
PPAR*γ*	CGCTGATGCACTGCCTATGA	AGAGGTCCACAGAGCTGATTCC
aP2	CATGGCCAAGCCCAACAT	CGCCCAGTTTGAAGGAAATC
ACC	GAATCTCCTGGTGACAATGCTTATT	GGTCTTGCTGAGTTGGGTTAGCT
CD36	GCTTGCAACTGTCAGCACAT	GCCTTGCTGTAGCCAAGAAC
SCD-1	TCACCTTGAGAGAAGAATTAGCA	TTC CCATTCCCTTCACTCTGA

**Table 2:** Sequence of the primers used in real-time PCR of HepG2 cell.

Gene	Forward primer	Reverse primer
*β*-Actin	AATCTGGCACCACACCTTCTA	ATAGCACAGCCTGGATAGCAAC
PPAR*γ*	TGCTGTATTTGAATCCGACGTT	GCTCTTTAGAAACTCCCTTGTCATG
aP2	GGGCCAGGAATTTGACGAA	GTACCAGGACACCCCCATCTAA
ACC	GGATGGTGTTCACTCGGTAATAGA	GGGTGATATGTGCTGCGTCAT
CD36	TGCTGTATTTGAATCCGACGTT	AAGGCCTTGGATGGAAGAACA
SCD-1	CCACCACATAGCATGCTTCCT	TGTTGCGGCATTGAATTAGC

## Abbreviations

ACC: Acetyl coenzyme A carboxylase
aP2: Adipose fatty acid-binding protein
CD36: Cluster of Differentiation 36
Cyp4a10: Cytochrome P450 4a10
Cyp4a14: Cytochrome P450 4a14
FAS: Fatty acid synthase
HDL-c: High density lipoprotein cholesterol
HF: High-fat
HFD: High-fat diet
HMGCR: HMG-CoA reductase
IPGTT: Intraperitoneal glucose tolerance test
LDL-c: Low density lipoprotein cholesterol
LXR: Liver X receptor
PGC1: Peroxisome proliferator-activated receptor gamma coactivator 1
PPAR: Peroxisome proliferator-activated receptor
SCD-1: Stearoyl-CoA desaturase-1
TC: Total cholesterol
TG: Triglyceride
WGP: Wax gourd peel.
